# Identification of nuclear localization signal and nuclear export signal of VP1 from the chicken anemia virus and effects on VP2 shuttling in cells

**DOI:** 10.1186/s12985-019-1153-5

**Published:** 2019-04-05

**Authors:** Jai-Hong Cheng, Guan-Hua Lai, Yi-Yang Lien, Fang-Chun Sun, Shan-Ling Hsu, Pei-Chin Chuang, Meng-Shiou Lee

**Affiliations:** 1 Center for Shockwave Medicine and Tissue Engineering, Department of Medical Research, Kaohsiung Chang Gung Memorial Hospital and Chang Gung University College of Medicine, 123 Tai-Pei Road, Niao Sung District, Kaohsiung, Taiwan 833; 2 Graduate Institute of Biotechnology, College of Agriculture and Natural Resources, National Chung Hsing University, Taichung, 40402 Taiwan; 3 Department of Veterinary Medicine, National Pingtung University of Science and Technology, Pingtung, Taiwan; 4 Research Center of Animal Biologics, National Pingtung University of Science and Technology, Pingtung, Taiwan; 5 Department of Bioresources, Da-Yeh University, Changhua, Taiwan; 6 Department of Orthopedic Surgery, Center for Shockwave Medicine and Tissue Engineering, Kaohsiung Chang Gung Memorial Hospital and Chang Gung University College of Medicine; Fooyin University, School of Nursing, Kaohsiung, Taiwan; 7 Department of Chinese Pharmaceutical Science and Chinese Medicine Resources, China Medical University, 91, Hsueh-Shih Road, Taichung, Taiwan

**Keywords:** Chicken anemia virus, VP1, VP2, Nuclear localization signal, Nuclear export signal, CRM1-dependent pathway

## Abstract

**Background:**

VP1 of the chicken anemia virus (CAV) is a structural protein that is required for virus encapsulation. VP1 proteins are present both in the nucleus and cytoplasm; however, the functional nuclear localization signal (NLS) and nuclear export signal (NES) of VP1 are still unknown. This study aimed to characterize the NLS and NES motifs of VP1 using bioinformatics methods and multiple-site fragment deletions, and investigate shuttling of VP2 from nucleus to cytoplasm by co-transfection with VP1.

**Methods:**

Two putative NLS motifs were predicted by the WoLF PSORT and NLStradamus programs from the amino acid sequence of VP1. Three NES motifs of VP1 were predicted by the NetNES 1.1 Server and ELM server programs. All mutants were created by multiple-site fragment deletion mutagenesis. VP1 and VP2 were co-expressed in cells using plasmid transfection.

**Results:**

A functional NLS motif was identified at amino acid residues 3 to 10 (RRARRPRG) of VP1. Critical amino acids 3 to 10 were significantly involved in nuclear import in cells and were evaluated using systematic deletion mutagenesis. Three NES motifs of VP1 were predicted by the NetNES 1.1 Server and ELM server programs. A functional NES was identified at amino acid residues 375 to 388 (ELDTNFFTLYVAQ). Leptomycin B (LMB) treatment demonstrated that VP1 export from nucleus to cytoplasm occurred through a chromosome region maintenance 1 (CRM1)-dependent pathway. With co-expression of VP1 and VP2 in cells, we observed that VP1 may transport VP2 from nucleus to cytoplasm.

**Conclusion:**

Our data showed that VP1 of CAV contained functional NLS and NES motifs that modulated nuclear import and export through a CRM1-dependent pathway. Further, VP1 may play a role in the transport of VP2 from nucleus to cytoplasm.

## Background

The chicken anemia virus (CAV) is a small virus of 23 to 25 nm in size; it is an icosahedral, non-enveloped virus, resistant to heat, lipid solvents and disinfectants [1]. CAV belongs to the genus Gyrovirus of the *Anelloviridae* family. The viral genome is a single-stranded DNA that is circular and covalently linked, consisting of approximately 2300 base pairs in its replicative form [2]. In 1979, Dr. Yuasa reported that vaccines contaminated with CAV had been distributed worldwide, causing spread of the disease [3]. CAV infection results in large economic damage, as the clinical disease contributes to vertical transmission and immune dysfunction in combination with other pathogens [4].

The CAV genome replicates through the rolling circle method [5]. The genomic DNA encodes three viral proteins (VP1, VP2 and VP3) from a 2.1-kb transcript [6]. Among the viral proteins, VP1 is a capsid protein (51 kDa) and VP2 is a non-structural protein (24 kDa), containing a dual-specific phosphatase activity and acting as a scaffold protein in the capsid assembly. VP1 and VP2 are protective proteins that induce neutralizing antibodies [7]. VP3 is called “apoptin”; it is the smallest of the three viral proteins at 13 kDa, with a unique apoptotic-inducing property [6, 8].

In the life cycle of the virus, the subcellular localization of viral proteins, especially in the nucleus, may contribute to cell apoptosis, viral replication or host cell proliferation [9, 10]. Thus, determination of the nuclear localization signal (NLS) and nuclear exporting signal (NES) of a viral protein is critical, and is required in order to reveal the importance of viral proteins in virus replication in cells. Recently, three CAV viral proteins, VP1, VP2 and VP3, have been found to possess nuclear localization activity in cells [11–14]. When GFP-VP1 and GFP-VP2 are transiently expressed in cells, VP1 and VP2 are observed to be present throughout the nucleoplasm [11]. VP3 has been reported to trigger cell apoptosis, and has been found to aggregate within the nucleus by phosphorylation of Threonine 108 [13, 14]. These results suggested that a functional NLS may be present in all three CAV viral proteins. To date, the functional NLSs of VP2 and VP3 have been demonstrated via bioinformatics and biochemical experiments [12–15]. VP3 contains a bipartite-type NLS and NES, suggesting potential shuttling of the protein between nucleus and cytoplasm [14, 15]. VP2 is a CRM1-independent nuclear protein with a simple NLS spanning amino acid residues from 133 to 138, and may not have a NES motif [12]. Moreover, VP2 and VP3 expressions have indicated protein–protein interaction in the cell [16]. Further, the apoptosis ability of VP3 is down-regulated directly by VP2 phosphatase through de-phosphorylation of Threonine 108 of VP3 [14]. VP1 is the only structural protein of CAV, and is required theoretically for packaging and formation of infectious particles [17]. A polyclonal chicken anti-CAV sera recognized all strains that have been tested, but only monoclonal antibody 2A9 has been mapped with VP1 [18–20]. The formation of epitopes recognized by polyclonal virus-neutralizing (VN) chicken antibodies requires co-expression of VP1 and VP2, implying that VP2 acts as a scaffold protein with VP1 [7, 21]. This finding was subsequently proved by analysis of VP1–VP2 protein–protein interaction [16]. In spite of this finding, the possible functional domain of VP1, especially in terms of the cellular localization, is not well understood, although some researchers have demonstrated the subcellular distribution of VP1 in cells using transient protein expression and immunofluorescence assays [11, 22]. The N-terminus of VP1 has been reported to possess a cell-penetrating activity [23]. Therefore, further research into and analysis of protein trafficking of VP1 may provide more information for elucidation of the biological function of nuclear localization of CAV viral proteins in virus replication.

In the present study, we used bioinformatics methods to predict putative NLS and NES motifs and identified the functional NLS and NES by performing experiments. Differing versions of VP1 were created by truncation mutagenesis, and the locations of functional NLS and NES sequences in VP1 were confirmed. Further, leptomycin B (LMB), an inhibitor of CRM1, was used to identify the function of the NES motif in the VP1 protein [15]. Finally, when VP1 and VP2 were co-expressed in cells, VP1 was observed to modulate the subcellular redistribution of VP2 from nucleus to cytoplasm. To the best of our knowledge, this is the first report to describe the functional NLS/NES motifs of VP1 and the interaction between VP1 and VP2 proteins allowing VP2 export from nucleus to cytoplasm.

## Methods

### Construction of multiple-site fragment deletions

For the expression of truncated VP1-GFP fusion proteins, the full-length VP1 plasmid (pGEX-6P-1-VP1) was used as a template to amplify fragments by polymerase chain reaction (PCR) with primers (Table 1) [24]. After agarose gel purification, PCR products were ligated into the yT&A vector (Yeastern Biotech, Taiwan). The PCR fragments were released from the yT&A vector by EcoRI/XhoI digestion and then inserted into pcDNA3.1-GFP [12].

**Table 1:** The primers were used to construct the various truncated, internal deletion mutants by PCR

Primer name	Type	Length	Sequence (5′-3′)
VP1 EcoR1	Forward	25-mer	AGAATTCATGGCAAGACGAGCTCGC
VP1 Xho1 R	Reverse	20-mer	TCCTCGAGGGGCTGCGTCTC
VP1 ND9 EcoR1	Forward	24-mer	AGAATTCATGGGCCGATTTTACGC
VP1 DN19 EcoR1	Forward	26-mer	AGAATTCATGCACAACCTCAAGCGAC
VP1 ND30 EcoR1	Forward	25-mer	AGAATTCAAATTTCGCCATCGCCGC
VP1 ND60 EcoR1	Forward	25-mer	AGAATTCCTGCCGAACCCGCAGAGC
VP1 ND129 EcoR1	Forward	25-mer	AGAATTCGGCGAACTGATTGCGGAT
VP1 CD10 Xho1	Reverse	21-mer	TCCTCGAGGCCTCTCGGTCTG
VP1 CD103 Xho1	Reverse	24-mer	TCCTCGAGGGCGACTCTCGGCCCC TAAGATGG
VP1 CD126 Xho1	Reverse	22-mer	TCTCGAGTCCGATTTTGCTCAC
VP1 internal NES deletion 1162	Forward	24-mer	GCGGCCGCGGCACAAATAAGTCGC
VP1 internal NES deletion 1119	Reverse	27-mer	GCGGCCGCATGATGCGGGTGCACT

### Analysis of amino acid sequence of VP1

Five isolates of CAV VP1, including Australia/CAU269–7/2000 (accession number: Q9IZU5), Germany Cuxhaven-1 (accession number: Q99153), Japan 82–2 (accession number: P54090), USA 26p4 (accession number: P54089), and USA CIA-1 (accession number: P54088), were identified in the Universal Protein Resource (UniProt). All sequences were aligned and analyzed using CLUSTAL W and MView in Biology Workbench 3.2 (San Diego Supercomputer Center; SDSC) [25, 26] and compared with Taiwan CIA-89. The putative NLS motifs of VP1 were predicted by WoLF PSORT [27] and NLStradamus [28], and the putative NES motifs were predicted by the NetNES 1.1 Server [29] and ELM server [30].

### Plasmid transfection

Chinese hamster ovary (CHO)-K1 cells were used for plasmid transfection. GIBCO® Dulbecco’s Modified Eagle Medium: Nutrient Mixture F-12 (DMEM/F-12) (Invitrogen, USA) was supplemented with 10% (*v*/v) fetal bovine serum (FBS) (GIBCO/Invitrogen, USA), 100 units/mL penicillin, and 100 μg/mL streptomycin for cell culture. A 24-well culture plate was seeded with 9 × 10^4^ cells per well for transfection. The plasmids were transfected into CHO cells using FuGENE® 6 transfection reagent (Promega, USA) according to the manufacturer’s instructions in order to observe the localization of VP1-GFP and various deletion mutants. For transfection of MDCC-MSB1 cells, 4 × 10^6^ cells were gently pipetted with 15 μg of pEGFP-VP1, pc-mCherry-vp2 and pEGFP-VP3 in serum-free RPMI 1640 medium separately. Each mixture was then transferred into a 0.4-cm gap electroporation cuvette, and the cuvette was harvested on ice for 5 min. Electroporation of MDCC-MSB1 cells was performed using a Gene Pulser II (Bio-Rad, USA) with a Time Constant Protocol set at 34 milliseconds and an operating voltage of 300 V. After electroporation, the transfected cells were cultured in complete medium in a 6-well plate for 24 to 48 h. Post-transfection, the expressions of recombinant EGFP-VP1, pc-mCherry-vp2 and pEGFP-VP3 proteins were analyzed by confocal fluorescence microscopy to ensure that transfection was effective.

### Leptomycin B treatment

CHO cells were seeded at a density of 9 × 10^4^ cells per well in 24-well culture plates for CRM1-dependent protein assay. In the experiments, cells were treated with fresh medium containing 20 ng/mL LMB (+) (Calbiochem, Germany) or with phosphate-buffered saline (PBS) as the LMB (−) control. After one hour of incubation, cells were fixed, washed and stained with DAPI [12]. Images of GFP fluorescence and DAPI were viewed and captured using a ZEISS AXIOVERT 200 microscope and an AxioCam HRm CCD camera, respectively.

### Sample preparation for confocal microscopy

Plasmids of pEGFP-VP1, pc-mCherry-vp2 and pEGFP-VP3 were constructed as described in a previous study [14]. MDCC-MSB1 cells were transfected with plasmids pEGFP-VP1, pc-mCherry-vp2 and pEGFP-VP3. Fluorescent images were captured using a confocal fluorescence microscope to observe protein fluorescence to verify the preparation of EGFP-expressing cells and EGFP-VP1, EGFP-VP3 and mCherry-vp2-expressing cells. Transfected cells were collected and fixed with 4% formaldehyde in the dark. After washing the fixed cells twice to remove residual formaldehyde, cells were stained in 0.1% PBS-T with 1 μg/mL DAPI for 5 min at 37 °C in the dark. Then, the stained cells were mounted with gelvatol medium (Sigma, USA) on a glass slide for confocal microscope observation. Confocal laser scanning microscope (CLSM) images were captured using a Leica TCS SP8 confocal microscope, and the images were integrated using LAS X Leica Confocal Software.

## Results

### Revisiting the distribution of VP1 in mammalian cells

**Fig. 1:**
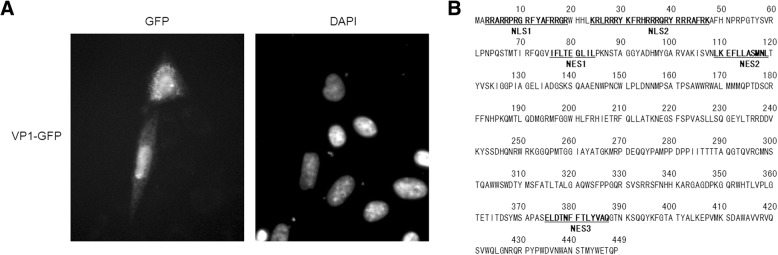
VP1 of CAV is distributed in the nucleus and cytoplasm. **a** CHO cells were transfected with VP1-GFP-expressing plasmids. At 48 h post-transfection, cells were fixed and stained with DAPI. The distribution of VP1-GFP in the cells was examined by fluorescence microscopy (600× magnification). **b** Prediction of NLS and NES motifs present in the VP1 amino acid sequence of Taiwan CIA-89 using bioinformatics methods employing WoLF PSORT and NLStradamus

In previous studies, VP1 has been shown to be distributed in the nucleus and cytoplasm, indicating that putative NLS and NES motifs of VP1 were functional in plant or chicken cells. To further revisit the subcellular localization of VP1, another cell line, CHO cells, was used to identify the NTS and NES of VP1. GFP on VP1 was constructed and applied as a reporter to track the distribution of VP1 in the cells. After transfection, VP1-GFP was observed to be located in the nucleus and cytoplasm of the CHO cells (Fig. 1a). This result clearly showed that possible functional NLS and NES might be present in VP1. Putative NLS and NES motifs were predicted using bioinformatics methods, as described in Methods (Fig. 1b).

### Characterization of NLS and NES motifs

**Fig. 2:**
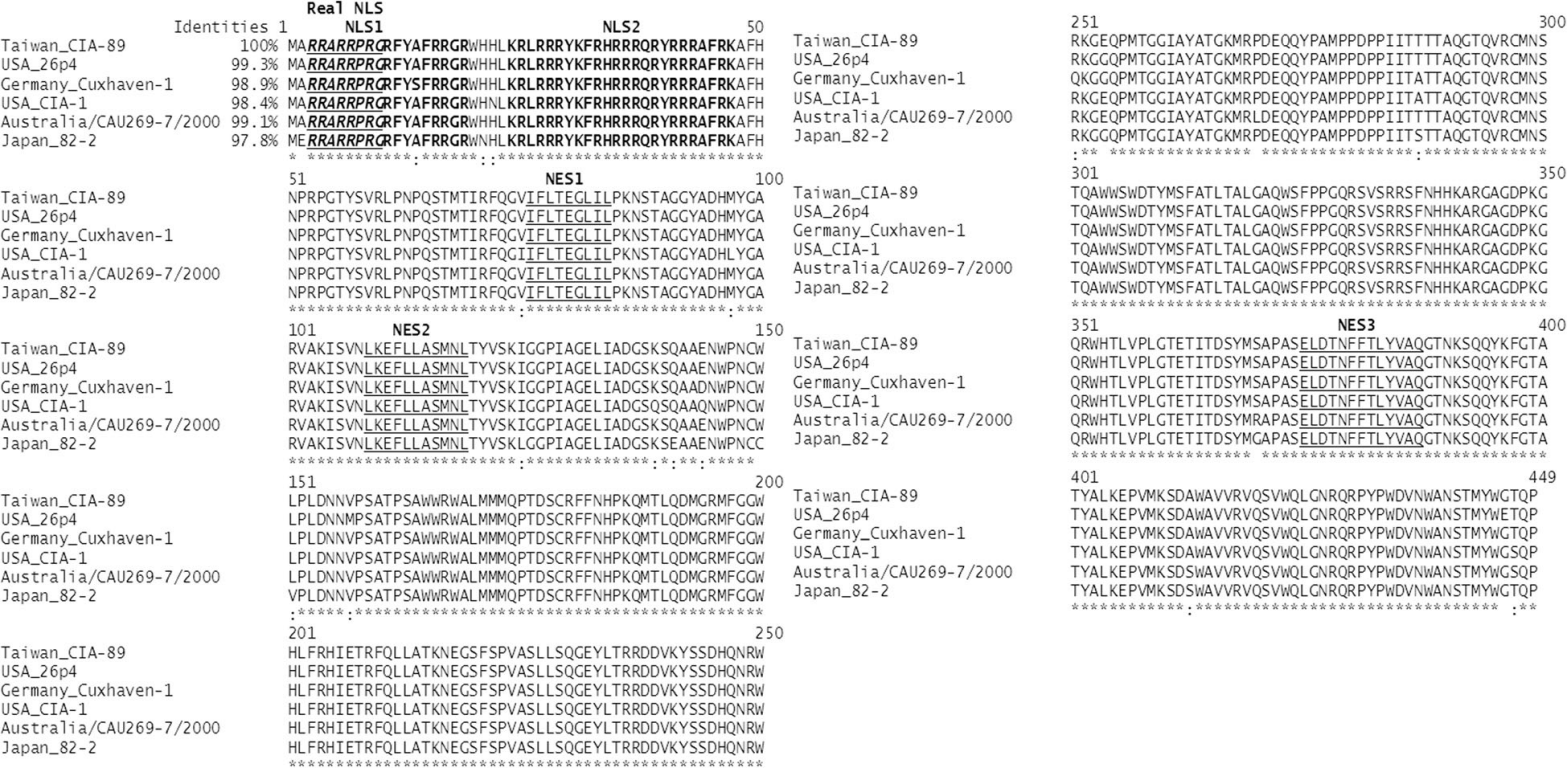
Alignment and prediction of NLS and NES motifs of VP1 present in different isolates of CAV. The various VP1 amino acid sequences (1 to 449) from different CAV isolates were aligned and their identities analyzed, as described in Methods. Putative NLS motifs were identified as spanning amino acids 3 to 19 (NLS1) and 24 to 47 (NLS2). Three putative NES motifs were found to be present, spanning amino acid residues 76 to 84 (NES1), 109 to 119 (NES2) and 375 to 387 (NES3). The functional NLS (Real NLS: amino acids 3 to 10) is shown and indicated in bold, italic and underlined

To identify potential NLS and NES motifs in VP1, the amino acid sequence of VP1 (Taiwan CIA-89) was compared with a range of other CAV isolates to reveal the protein sequence divergence. The various VP1 sequences of the different CAV isolates were obtained and sequence alignment performed to demonstrate that all CAV isolates were highly-conserved (Fig. 2). The identity matches were 99.3% (USA 26p4), 99.1% (Australia/CAU269–7/2000), 98.9% (Germany Cuxhaven-1), 98.4% (USA CIA-1) and 97.8% (Japan 82–2) as compared with Taiwan CIA-89.

Computational prediction of protein localization from the amino acid sequence was an important step in the present study. Numerous computational methods are available for analysis of the protein sequence to predict the most likely locations, such as in the nucleus or in the cytoplasm. Therefore, we used the WoLF PSORT and NLStradamus programs to predict the NLS sequence from the full-length amino acid sequence of VP1 (Taiwan CIA-89) (Figs. 1b and 2). Two NLS motifs (designated NLS1 and NLS2) were predicted by the WoLF PSORT program. The putative NLS motifs spanned amino acid residues from 3 to 19 (NLS1) and 24 to 47 (NLS2). On the other hand, putative NES motifs were identified by the NetNES 1.1 Server and ELM server (Fig. 2). Three putative NES motifs spanning amino acid residues 76 to 84 (NES1), 109 to 119 (NES2) and 375 to 387 (NES3) were pinpointed. In light of these results, further investigations were needed in order to elucidate whether the putative NLS and NES were functional in VP1.

### Functional NLS of VP1

**Fig. 3:**
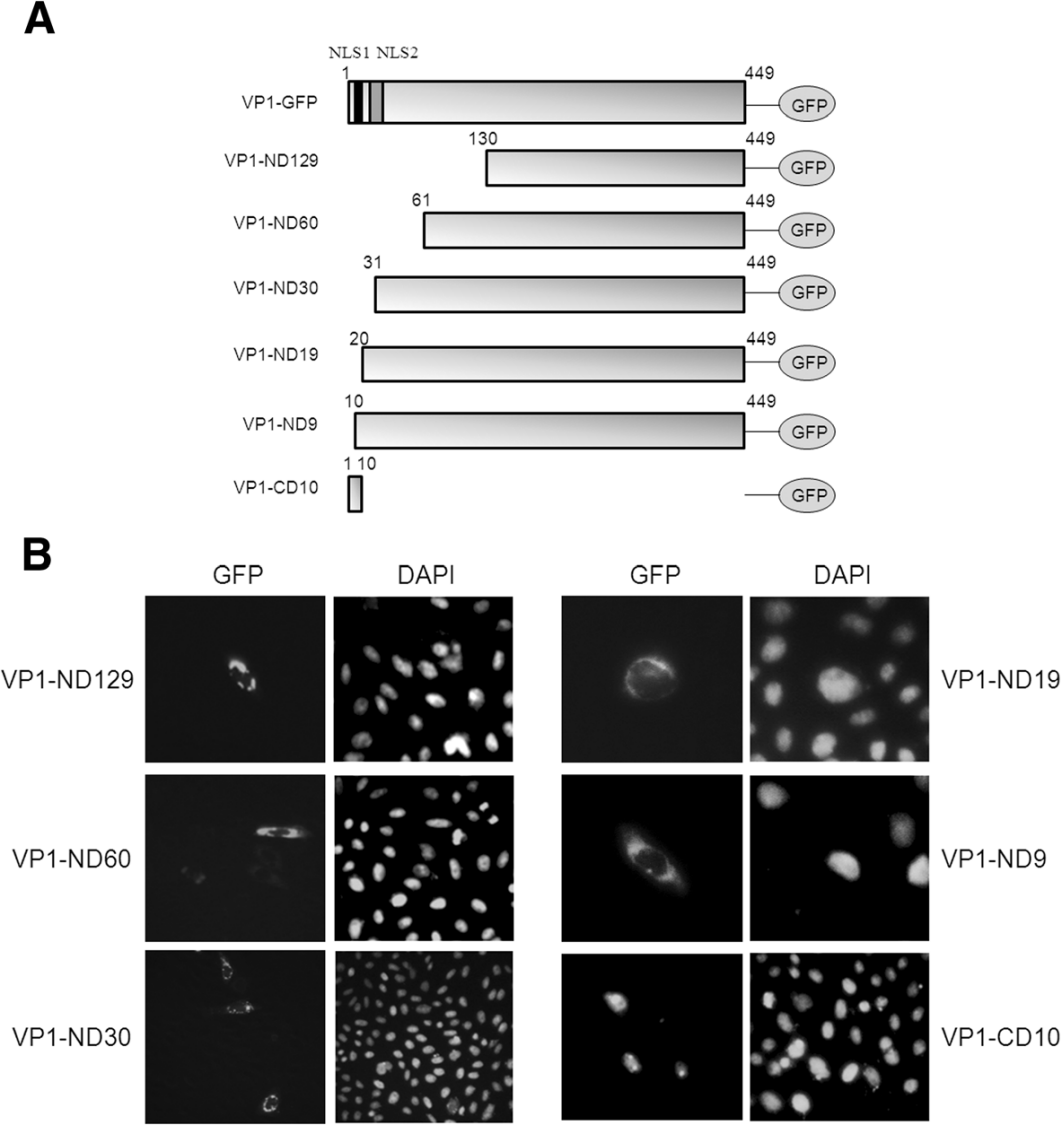
NLS motif characterized by multiple-site fragment deletions. **a** Truncated fragments of VP1 (gray bars) encoding constructs fused with GFP (oval shape) were used in this study, including VP1-ND129, VP1-ND60, VP1-ND30, VP1-ND19, VP1-ND9 and VP1-CD10. The putative NLS1 (black box) and NLS2 (gray box) are shown in the full length of VP1 (gray bar). The numbers of amino acid residues are presented in the constructs. **b** Subcellular localization of N-terminal deleted VP1-GFP constructions (VP1-ND129, VP1-ND60, VP1-ND30, VP1-ND19 and VP1-ND9) and C-terminal deleted VP1-CD10 are shown in CHO cells. All cells were fixed and stained with DAPI. The distributions of the truncated VP1-GFP constructs in the cells were observed using fluorescence microscopy (400× magnification)

Using bioinformatics analysis, two putative NLS motifs (NLS1 and NLS2) were predicted to be present in the N-terminal of VP1 (Fig. 2). Hence, in order to determine the functional site of the NLS motif in VP1, a full-length clone and six deletion clones of VP1 were created to fuse with GFP at the C-terminal (Fig. 3a). The subcellular locations of these expressed constructs were examined in the transfected cells based on the GFP distribution patterns at 48 h post-transfection. The serious truncated N-terminal deletions of VP1-GFP identified for NLS analysis were VP1-ND129, VP1-ND60, VP1-ND30, VP1-ND19 and VP1-ND9. The green fluorescence of these truncations was predominantly distributed in the cytoplasm of CHO cells (Fig. 3b). These results suggested that the NLS motif of VP1 was monopartite, presented between the 3rd and the 9th amino acid in the sequence. Furthermore, VP1-CD10 was tested, and the GFP was found to be localized in the nucleus of CHO cells (Fig. 3b).

### NES motif in VP1

NES in capsid protein VP1 of the CAV positive-strand DNA virus is not well-characterized; nor is the pathway that determines its export from nucleus to cytoplasm. Therefore, we characterized the computational prediction of NES motifs within VP1. Three putative NES motifs were identified, spanning amino acid residues 76 to 84 (NES1), 109 to 119 (NES2) and 375 to 387 (NES3) in VP1 (Fig. 1b and Fig. 2).

**Fig. 4:**
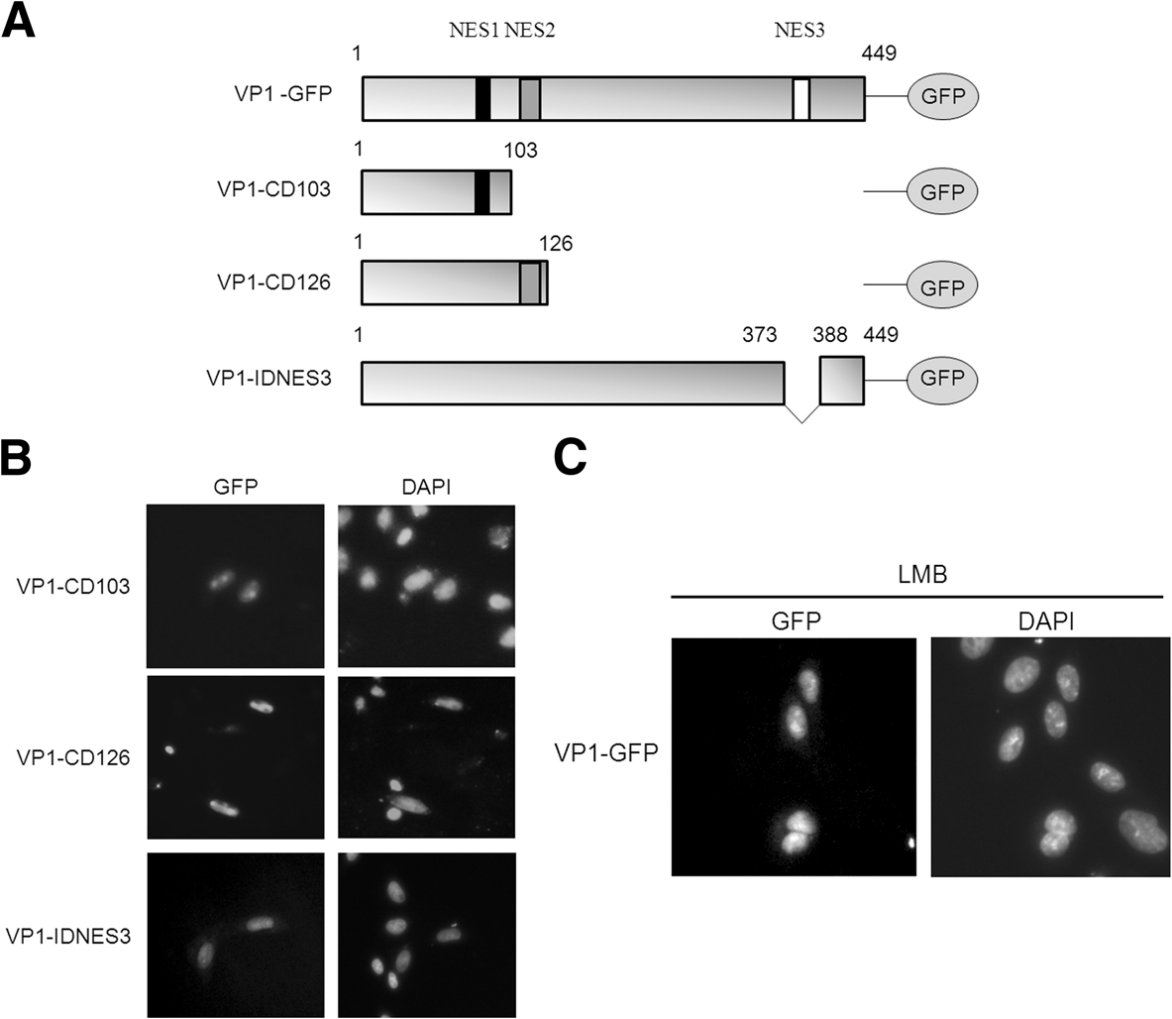
The NES motif of VP1 was characterized and found to be CRM1-dependent for nuclear export to the cytoplasm. **a** Truncated constructions of VP1-GFP were used to identify the NES motif. Truncated fragments of VP1 (gray bars) encoding constructs fused with GFP (oval shape) were used in this study, including VP1-CD103, VP1-CD126 and VP1-IDNES3. The putative NES1 (black box), NES2 (gray box) and NES3 (white box) are shown in VP1 (gray bars). The numbers of amino acid residues are presented in the constructs. **b** Subcellular localization of C-terminal deleted VP1-GFP constructions (VP1-CD103 and VP1-CD126) and internal deleted VP1-IDNES3 are shown in CHO cells. All cells were fixed and stained with DAPI. **b** VP-GFP was treated with LMB (20 ng/mL) after 48 h of transfection. The distributions of the wild-type VP1-GFP and the truncated VP1-GFP in the cells were observed using fluorescence microscopy (400× magnification)

In order to verify functionally-significant NES motifs, we created various truncated mutants of VP1-GFP (Fig. 4a). After transfection, we observed that the C-terminal-truncated mutants (VP1-CD103 and VP1-CD126) were distributed in the nucleus (Fig. 4b). This result indicated that NES1 and NES2 were not NES motifs and could not assist in truncated VP1 export to the nucleus. The results also showed that the N-terminal region of VP1 (amino acid 1 to 126) did not contain an NES motif. Further, we created an internal deletion mutant, VP1-IDNES3, to verify the function of NES3 (Fig. 4a and b). VP1-IDNES3 was located in the nucleus after transfection. We compared the distribution of VP1-GFP and VP1-IDNES3 in the cells, and the results suggested that NES3 was the NES motif (Fig. 4b).

### VP1 is a CRM1-dependent protein

Typically, the nuclear protein containing NES motifs can shuttle between nucleus and cytoplasm. In order to evaluate the functional NES3 motif, we used LMB to interfere with CRM1–NES interaction and verified the functionality of the NES motif (Fig. 4c). Functional NES3 was investigated using VP1-GFP, as shown in Fig. 4c, together with LMB (+) (20 ng/mL). The distribution of green fluorescence was observed, and the results demonstrated that LMB was able to affect the nuclear export of VP1. Therefore, it was evident that VP1 nuclear export was a CRM1-dependent process and that there was a functional NES motif present in VP1.

### Nucleo-cytoplasmic shuffling of VP2 is triggered by VP1

**Fig. 5:**
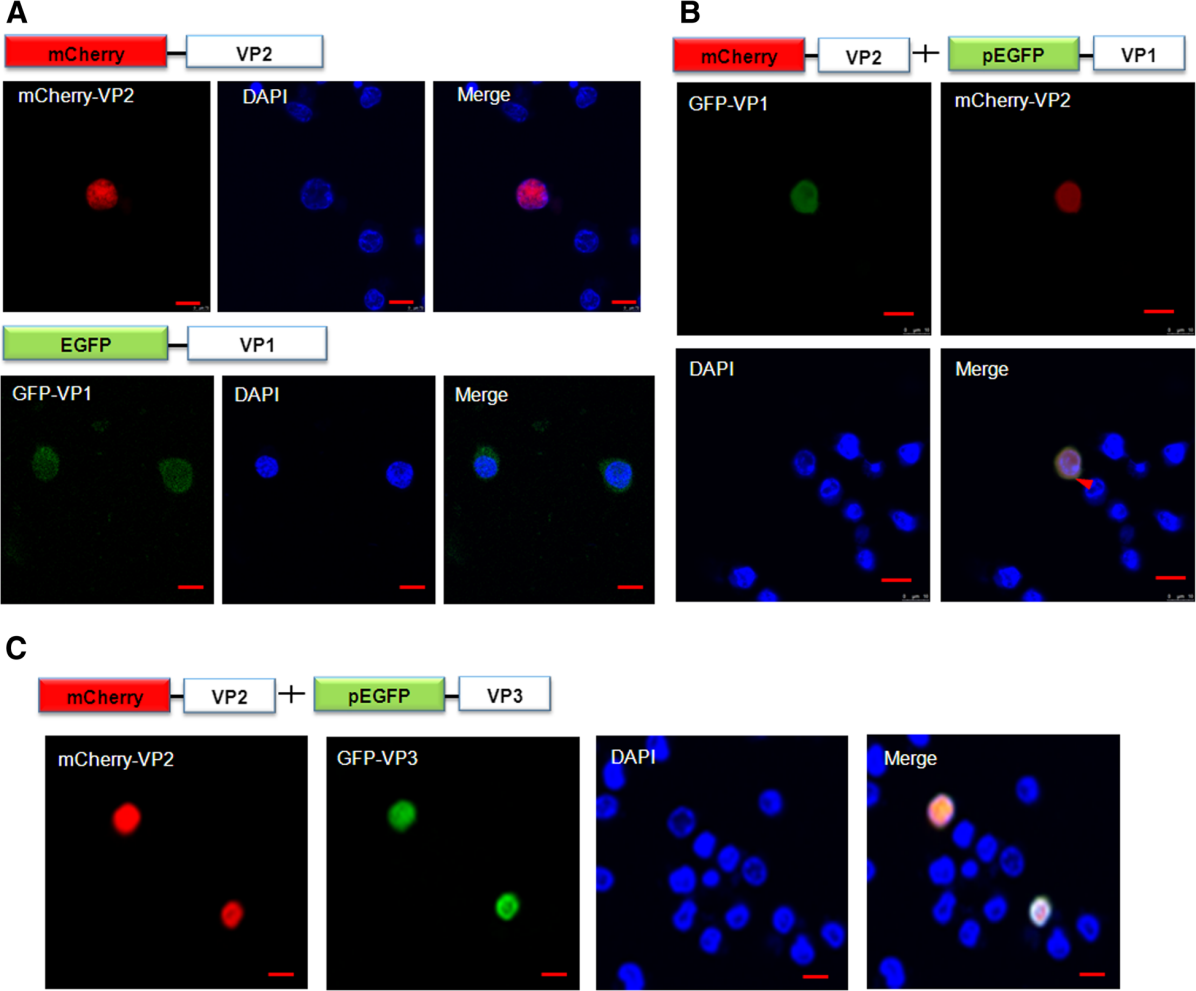
VP1 modulated the export of VP2 from nucleus to cytoplasm in MDCC-MSB1 cells. **a** Over-expression of VP2 (red) was seen in the nucleus, and VP1 (green) was distributed between the nucleus and cytoplasm. **b** Co-expression of VP1 (green) and VP2 (red) was observed in the cells. VP2 was exported by VP1 from the nucleus into the cytoplasm (indicated by a red arrow head). **c** VP2 (red) was not exported by VP3 (green) in the cells. Scale bar = 10 μm

In a previous study, VP2 exhibited nuclear localization activity and did not contain an NES motif. In contrast, when VP1 was transiently-expressed in MDCC-MSB1 cells, the subcellular distribution of VP1 was observed to be in the nucleo-cytoplasmic compartment, and this result was consistent with the subcellular localization of VP1 in CHO cells (Figs. 1 and 5a). Notably, however, VP2 was exported from the nucleus into the cytoplasmic compartment when transient co-expression of VP2 and VP1 was attained, but not with co-expression of VP2 and VP3 (Fig. 5b and c). Further, the subcellular distribution of VP1 did not change in MDCC-MSB1 cells when VP2 was co-expressed at the same time. Taking these results together, VP1 displayed an interaction with VP2 in terms of co-localization of VP1 and VP2 in cells, and VP2 export from nucleus to cytoplasm was triggered by VP1.

## Discussion

Young chickens infected with CAV suffer anemia, hemorrhaging, and immunosuppressive diseases, and the mortality of the chickens is increased [1, 3, 31]. The virus contains a very compressed genomic organization, and three viral proteins (VP1, VP2 and VP3) translated from a single polycistronic 2.1-kb mRNA have been identified in virus-infected cells [3, 6]. VP1 is the only structural protein that has a very basic N-terminal region that encapsulates a negative-strand DNA genome [32]. The C-terminal region of the protein carries a conserved motif for rolling circle replication (RCR) [33, 34]. In this study, CAV VP1 was observed in the nucleus and cytoplasm in transfected CHO and MDCC-MSB1 cells (Figs. 1 and 5). Sequence analysis indicated that the N-terminus of the CAV VP1 protein contains abundant basic amino acid residues (Fig. 2). Investigation of the subcellular localization of the truncated CAV VP1 proteins fused with GFP indicated that the 7 amino acids at the N-terminus were crucial and sufficient to direct the accumulation of the protein in the nuclei (Fig. 3), and amino acid residues from 375 to 387 exhibited a functional NES motif (Fig. 4). Dr. Hu reported that the N-terminus of the VP1 protein consists of enriched arginine residues similar to HIV-TAT [23]. The sequence has an efficient cell-penetrating activity. However, that study did not identify the sequence systemically to show that it is the NLS of VP1. The other viral protein, VP3, is a nucleocytoplasmic shuttling protein, the localization of which is mediated by an N-terminal NES and a C-terminal NLS [13, 15]. Although CAV VP1 and VP3 were shuttled between nucleus and cytoplasm during virus infection, VP2 was restricted to the nuclei in plasmid-transfected cells [12].

In addition, VP1 interacts with VP2, which is a non-structural protein exhibiting phosphatase activity, and the dual specificities are indispensable for CAV replication [21, 35–37]. VP2 has been found to contain a functional NLS and is a CRM1-independent protein [12]. Further, VP2 also associates with mini-chromosome maintenance protein 3, a component of DNA pre-replication complexes [12]. In this study, under co-transfection of VP1 and VP2, it was observed that VP2 might be shuffled by VP1 between nucleus and cytoplasm in cells. Dr. Sun reported that a specific interaction domain between VP1 and VP2 was observed by yeast two-hybrid system [16]. As VP2 lacks an NES motif, VP1 may act as a nuclear exporting factor with VP2 for nucleo-cytoplasmic shuffling in cells. There may be other hosts involved in the export of VP2 by VP1 from nucleus to cytoplasm for regulation of the viral life cycle or hosting cell proliferation. Molecules that interact with VP2 in the cytosol require further investigation in the future.

NES is a short, leucine-rich motif that is an export substrate of CRM1 and identifies in HIV Rev. and protein kinase A inhibitor [38, 39]. The function of NES is important for the virus during the viral life cycle and plays a vital role in the replication, assembly, and budding of viruses. For example, the influenza A virus matrix 1 protein (M1) is specifically dependent on the Flu-A-M1 NES and is critical for influenza A virus replication [40]. CRM1-mediated nuclear export has been reported to be involved in several viral infections, including the influenza A virus [40, 41], human papillomavirus [42] and HIV [38, 43]. Our data indicated that CAV VP1 utilized a CRM1-dependent nuclear export pathway, as well as VP3 (Fig. 4b). For the previously-identified NES, only two leucines located in VP3 contributed to the activity of this motif [44]. Only two leucines (Leu376 and Leu383) have been discovered in the VP1 NES, and a number of leucine-rich NESs have been characterized, in which one or two leucine residues contribute to the nuclear export activity. Taken together, the nucleocytoplasmic shuttling activity suggested that VP1 may coordinate with other viral proteins such as VP2 and VP3 to complete the viral life cycle.

## Conclusion

In the current study, VP1 of CAV was found to contain a functional NLS motif that spanned amino acids 3 to 9 of the protein. Further examination showed that VP1 also has an NES motif spanning amino acids 375 to 387 at the C-terminus. VP1 exhibited sensitivity to the CRM1 inhibitor LMB, and was found to be a CRM1-dependent protein. Characterization of the NLS and NES of the CAV VP1 protein provided the basis for further research. This study also explored the role of nuclear involvement of the VP1 protein in the development of CAV infection and the biological implication of its shuttling between nucleus and cytoplasm.

## Abbreviations

CAV: Chicken anemia virus
CHO: Chinese hamster ovary
CRM1: Chromosome region maintenance 1
DMEM/F-12: Dulbecco’s Modified Eagle Medium: Nutrient Mixture F-12
FBS: Fetal bovine serum
LMB: Leptomycin B
NES: Nuclear export signal
NLS: Nuclear localization signal
PBS: Phosphate-buffered saline
RCR: Rolling circle replication
